# Classification of 5‑bit Binary Light Pulse Sequences Using Photoluminescence of Metal Halide Perovskite Memlumors

**DOI:** 10.1021/acsenergylett.5c01369

**Published:** 2025-07-11

**Authors:** Jitendra Kumar, Daniel Lizotte, Alexandr Marunchenko, Dmitry Tatarinov, Shivam Singh, Yana Vaynzof, Ivan. G. Scheblykin

**Affiliations:** † Chemical Physics and NanoLund, 5193 Lund University, 22100 Lund, Sweden; ‡ School of Physics and Engineering, 65071 ITMO University, St. Petersburg 197101, Russian Federation; § Chair for Emerging Electronic Technologies, 9169 Technical University of Dresden, 01187 Dresden, Germany; ∥ 28394 Leibniz-Institute for Solid State and Materials Research Dresden, 01069 Dresden, Germany

## Abstract

A memlumor is an innovative neuromorphic luminescent device with a state-dependent photoluminescence quantum yield (PLQY) designed for optical neuromorphic computing applications. Metal halide perovskite memlumors leverage charge trapping and photodoping to modulate the PLQY, making it dependent on the excitation light history. Here, we demonstrate the ability of perovskite memlumors to classify time-dependent binary optical signals on sub-microsecond timescales. Sequences of laser pulses (representing ones) and gaps (absence of pulses representing zeros) were used to excite photoluminescence in MAPbBr_3_ and triple cation perovskite films. By reading only the time-integrated PL signal, we completely recognized the time-dependent input patterns containing 5 bits of information. The potential of perovskites for applications in optical reservoir computing based on their complex and diverse photophysics is discussed.

## Graphical abstract

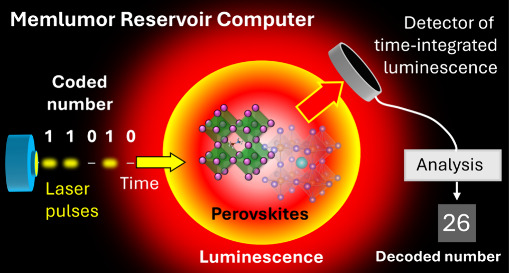

The rapid increase in computational demands has driven the exploration of new methods and materials for energy-efficient neuromorphic optical computing.^1–8^ Various photonic circuits using light diffraction and interference to create neural networks or their parts have been proposed.^9–15^ Recently, it has been recognized that light emitted by a material in response to optical excitation (input) has significant potential for optical computations: it can hold the memory of the input signal and transform time-dependent input in a complex, nonlinear manner. Devices leveraging this idea are referred to as memitters (memory + emitter) or memlumors (memory + luminophore) in the literature, representing a novel class of systems capable of utilizing the phenomenon of light emission for computing.^16–25^

Photoluminescence (PL) is a well-known phenomenon in which excitation light creates excited states in the material (e.g., excitons, free charge carriers, etc.), which release some of their energy by emitting photons of slightly lower energy. Materials exhibiting this behavior are often called fluorophores or, more generally, luminophores. For most luminophores, PL intensity scales linearly with excitation power density, meaning that the photoluminescence quantum yield (PLQY) is independent of the excitation power density and its temporal history. Even this straightforward input-output relationship has potential applications in computing. Since PL has a finite lifetime, it can serve as volatile memory, with the PL signal persisting long after the excitation pulse (ranging from nanoseconds to minutes). A simple example is phosphorescence, where materials emit PL light for extended periods (seconds to minutes) in response to short (μs-ms) excitation pulses. This property of PL can be utilized for time-averaging of time-dependent optical signals. However, if PLQY of a system is not constant and depends on the excitation light and its temporal history, the system becomes a memlumor with a much higher potential for computational applications.^17,20^

Applying the terminology accepted for electrical memristors to memlumors, we can say that the PLQY of a memlumor depends on the time-dependent state vector X (which contains the information on excited states created by the excitation light), and PLQY is a transfer function between PL­(t) and excitation intensity I­(t): PL(t) = PLQY(X(t))·I(t).^17^ This formalism enables memlumors to be analyzed using the same mathematical framework as memristors, with PLQY­(X­(t)) for a memlumor serving an analogous role to the state-dependent conductance for a memristor.^17,26^

The effect of photodoping in semiconductors is probably the simplest mechanism of memory in PLQY.^16,27^ It was shown that the PLQY of a luminescent semiconductor is modulated by several internal variables leading to the photodoping effect, as evidenced in metal-halide perovskites (MHP) semiconductors.^16,17^ For instance, CsPbBr_3_ perovskite memlumors were shown to possess neuromorphic features such as pair-pulse facilitation (PPF), potentiation, and hysteresis across multiple time scales.^16,17^

One of the driving ideas behind new computational architectures for artificial neural networks is to emulate processes occurring in biological brains.^28^ Unlike binary logic in digital systems, the brain operates using temporal signals in the form of electrical spikes, where timing, amplitude, and shape - all contribute to information processing.^29,30^ This represents a shift from purely digital to more analogue forms of computing. A system capable of converting time-dependent digital optical signals into an analogue signal containing ciphered information about the temporal sequence of the input is highly desirable for the operation of optical neural networks.^31^

In this work, we propose and demonstrate that MHP memlumors can classify time-dependent binary sequences by leveraging charge trapping and photodoping effects. Binary information is encoded into laser pulses with precise timing and directed at a MHP film. The material’s internal state, influenced by the timing and intensity of the pulses, generates a unique PL response. A photodetector reads the time-integrated PL signal, which depends not only on the number of pulses but also on their temporal arrangement (Figure 1). We report the experimental realization of a 5-bit sequence classification system using MAPbBr_3_ and triple cation perovskite films as memlumors, highlighting their potential for optical neuromorphic computing.

**Figure 1:**
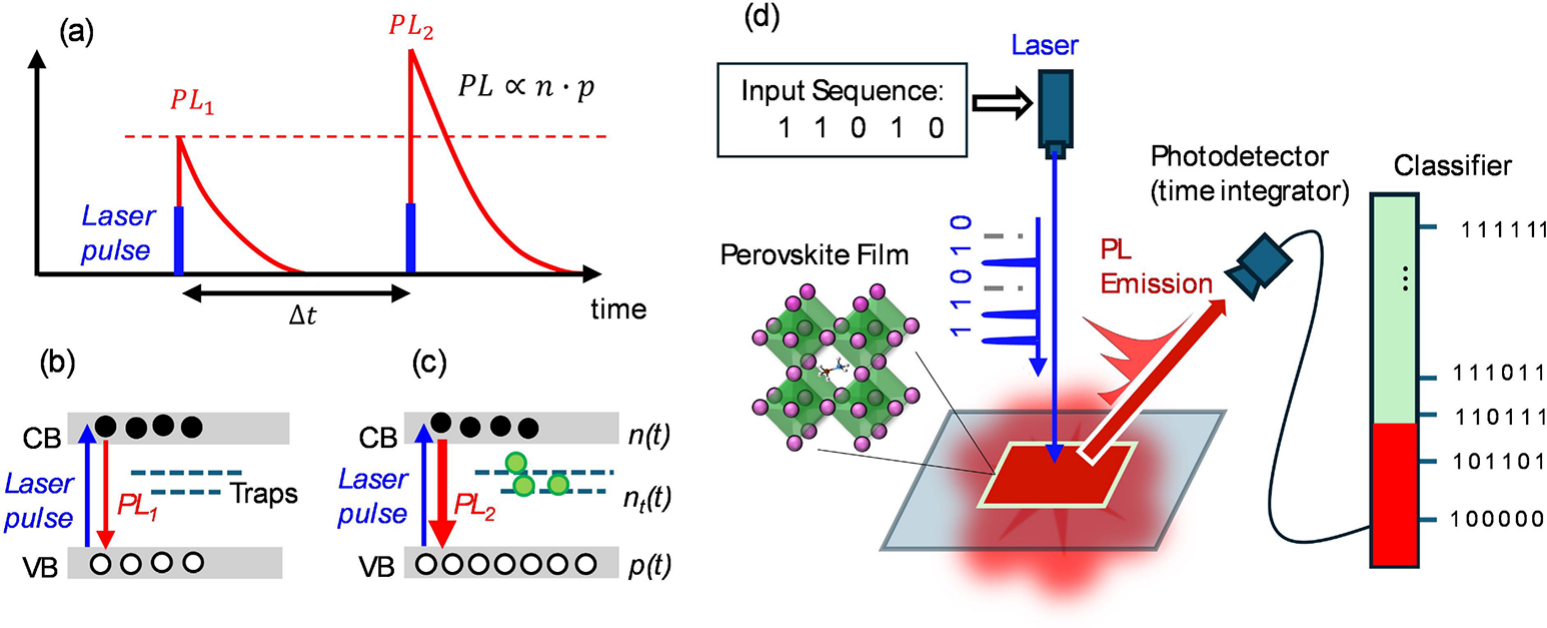
Schematic diagram of the use of memlumors for decoding information from time-dependent input signals. a) For a memlumor, the PL response on two excitation pulses depends on the time gap between the pulses, reflecting the combined effects of nonlinearity and memory. b,c) Band diagram of the memlumor excited by b) the first pulse and c) the second pulse at the moment of the pulse arrival. The presence of traps leads to the photodoping effect (more free holes in the system when the second pulse arrives), which makes PL_2_ > PL_1_. d) The experimental setup scheme for binary code classification by time-integrated PL of a metal halide perovskite film.

One of the origins of memory effects in the electronic or optical response of semiconductors is a population of long-living defect states.^16,27,32,33^ Let us consider a semiconductor possessing defect states with levels close to one of the bands, for example, the conduction band (Figure 1a-c). In this case, the probability of electron trapping will be high, while recombination of the trapped electron with a free hole is rather slow. Under light illumination, this will lead to a buildup of a substantial concentration of trapped electrons *n*_*t*_. Due to the charge neutrality condition *n* + *n*_*t*_ = *p*, the concentration of free electrons becomes smaller than that of free holes - the p-type photodoping effect. Since PL occurs due to recombination of a free electron and a free hole, its intensity is proportional to the product *n* · *p*. Thus, increasing the concentration of one of the charge carriers (like p-photodoping in our example) gives a higher PL intensity for the same concentration of the opposite charge (see Figure 1c). Photodoping leads to PPF, which enhances the time-integrated PL response of the system to two identical input pulses if the time delay between them decreases. It means that the PLQY depends on the distance between two pulses (Figure 1a). PPF is shared by biological neurons^34–36^ and is a signature of a neuromorphic device.

If the PL response depends on the distance between two pulses, then upon excitation by a burst of N pulses, the PL response should depend not only on the number N but also on the temporal separation between all individual pulses. This may enable decoding of binary pulse sequences using time-integrated PL as an output signal. Let us imagine we can send a pulse burst of 5 equally separated pulses. This corresponds to the binary sequence ‘11111’ or the number 31. By skipping some pulses, we can obtain any 5-bit digital sequence. However, it is not obvious that all these sequences will generate integrated PL responses sufficiently different from each other to be uniquely identified in real experiments.

It is important to note that the first pulse (denoted as “1” in the leftmost position) must always be present, as it serves as the marker indicating the start of the sequence. Without this marker, it is not possible to differentiate between 5-bit sequences that appear identical but shifted in time (e.g. “00110”, “01100”, and “11000”). So, to distinguish them, we add one pulse to the left (the marker), which makes the sequences for these mentioned examples to be “**1**00110”, “**1**01100”, and “**1**11000”. It is equivalent to sending a 6-bit code, but using for actual coding only the last 32 numbers: from 32 (“**1**00000”) to 63 (“**1**11111”).

To verify this idea, we modeled the PL responses of a semiconductor possessing photodoping to such excitation sequences. The only difference to Figure 1 and the description above was that due to technical issues of the experiment (see below) it was not practical to code “1” by one pulse, and “0” by an absence of a pulse. Instead, we used a burst of 10 pulses as “1” and the absence of 10 pulses as “0”. The same excitation coding (Figure S2) was applied in the modelling by an extended Shockley-Read-Hall model described in previous publications in detail.^16,27^ Section 3 of the Supporting Information (SI) contains the equations of this model and the used model parameters.

Figure 2 shows the modeling results for two rather different memlumors (different sets of model parameters), which represent typical situations observed in various MHP systems.^16,27,32,37^ The PL response here is the time-integrated signal illustrated in Figure 1. As expected, most of the different sequences give rather different responses. The most significant difference is observed for the sequences with different number of “1”. This difference is trivial and should be observed for any luminophore, even with a constant PLQY (for example, a dye solution). However, when the numbers of "1" are the same, the differences are still present (due to PLQY­(X­(t)) ≠ const), but much less pronounced and, for some pairs, are very small. To quantify and illustrate this problem, we calculated the separation matrix (Figure 2c,d), a plot comparing the PL response for one sequence (*X*-axis) with the PL responses for all other sequences (*Y*-axis). Each value on this plot represents the relative separation δ = 100% × | S(code1) – S(code2) |/S(code1), where S­(code1) and S­(code2) are PL outputs generated by excitation sequences code1 and code2. The labels of the X and Y axes on the figure are the decimal equivalents of the coded numbers ranging from 32 to 63.

**Figure 2:**
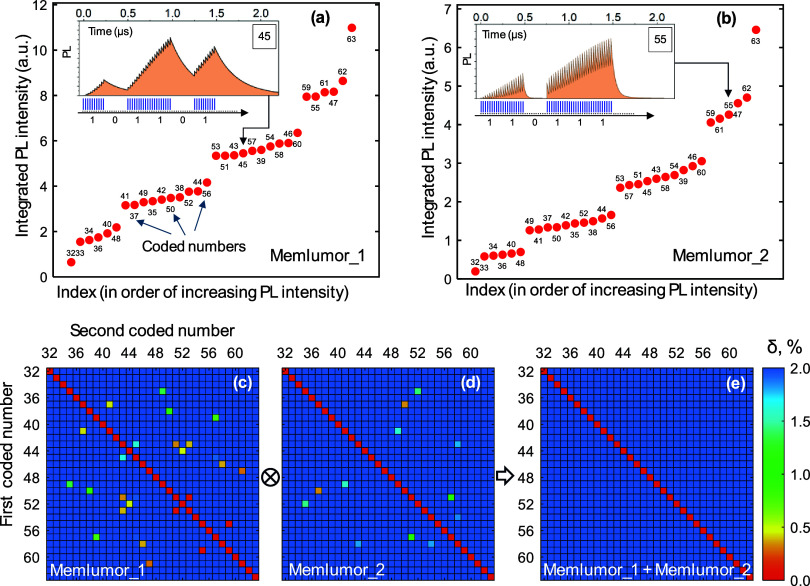
Calculated PL responses of two different memlumors (Memlumor_1 and Memlumor_2, which have different Shockley-Read-Hall model parameters) to binary excitation pulse burst sequences (codes) corresponding to the decimal numbers from 32 to 63. a) and b) - integrated PL signal for different binary sequences arranged in ascending order of the time-integrated PL intensity. Insets in a) and b) show examples of time-resolved PL responses for two memlumors to the excitation sequences corresponding to the numbers 45 (a) and 55 (b). Realization of these sequences using bursts of laser pulses is illustrated: blue lines – laser pulses, the dotted line shows the 40 MHz clock (*T*_0_ = 25 ns between the dots). c, d) The separation matrices for both memlumors. e) A combined separation matrix when two memlumors are used to solve the task simultaneously. All coded numbers are separated from each other by at least 2%.

We are interested in comparing different sequences, so the diagonal is irrelevant to the analysis. All separations >2% are considered sufficient (shown in blue), while lower separations are considered insufficient and illustrated according to the red-orange color code. While there are codes with insufficient separation for each calculated memlumor on its own (Figure 2c,d), these codes are different for different memlumors (compare Figure 2c and Figure 2d), suggesting that by combining several different memlumors, a sufficient separation (complete decoding) should be possible. The difference in the response of two memlumors is further illustrated in Figure S6a.

When two memlumors are used, the separation occurs in the 2-dimensional (S_1_,S_2_) space, where the outputs for code1 and code2 have the coordinates (S_1_(code1), S_2_(code1)) and (S_1_(code2), S_2_(code2)), see SI section 6. Then the relative separation becomes $\delta_{1+2}=\sqrt{\delta_{1}^{2}+\delta_{2}^{2}}$ . The combined separation matrix calculated in Figure 2e shows no combinations of codes with the separation less than 2%, thus demonstrating a sufficiently accurate decoding of the entire 5-bit information sequence. It is additionally illustrated in the SI in Figure S4 where signals for all codes are plotted in the two-dimensional (S_1_,S_2_) space.

To realize this concept in practice, we choose two perovskite films: MAPbBr_3_ and triple cation perovskite (Cs_0.05_(FA_5/6_MA_1/6_)_0.95_Pb­(I_0.9_Br_0.1_)_3_) because of the substantial difference in their characteristic PL lifetime and charge dynamics.^16,32^ The preparation of these samples is described in detail in section 1 in the SI. A 485 nm picosecond diode laser (PicoQuant) was used for excitation, while a SEPIA 828D laser pulse controller (PicoQuant) was used to program and send any sequence of ∼ 200 ps long pulses to the sample.^38^ In our experiments 40 MHz base clock was used, meaning that we could choose to fire or not a laser pulse each 25 ns using the SEPIA controller. More details can be found in section 2 of the SI and in our recent publication.^38^

Due to a technical issue related to the stability of the relative amplitude of the pulses in the pulse burst mode, it was not possible to use just a single pulse as “1” in the binary code, instead, a burst of 10 pulses (*T*_0_ = 25 ns separation between neighboring pulses) was used. Similarly “0” was a repetition of 10 periods *T*_0_ without firing pulses; see SI sections 4, 5 and Figure S2. Each input sequence lasted 6 × 10 × 25 ns = 1.50 *μs,* and it was repeated every 8 ms. Such a long repetition period was needed for the memory created by one sequence to decay before the same sequence excites the sample again. The PL intensity (output) was integrated over the exposure time (100 ms or longer) of the CCD camera used as a detector.

Figure 3 shows the experimental results for each of the perovskite films. The insets show the actual readings of the CCD camera in response to the sequentially applied excitation light encoding each binary number from 32 (code "100000") to 63 (code "111111"). The laser pulse controller was programmed to send each coded excitation pattern for 10 s. After that, the laser was off for 1 s, and during this pause, the settings of the controller were changed to the next sequence, and then the next code was sent. Figure 3a, b, show the signal averaged over 10 s and sorted in the order of its increase for both memlumors. As predicted by the modeling described above, the separation between the codes that contain different number of “1” (2,3,4,5,6) is large, while the separation within each such group is more subtle.

**Figure 3:**
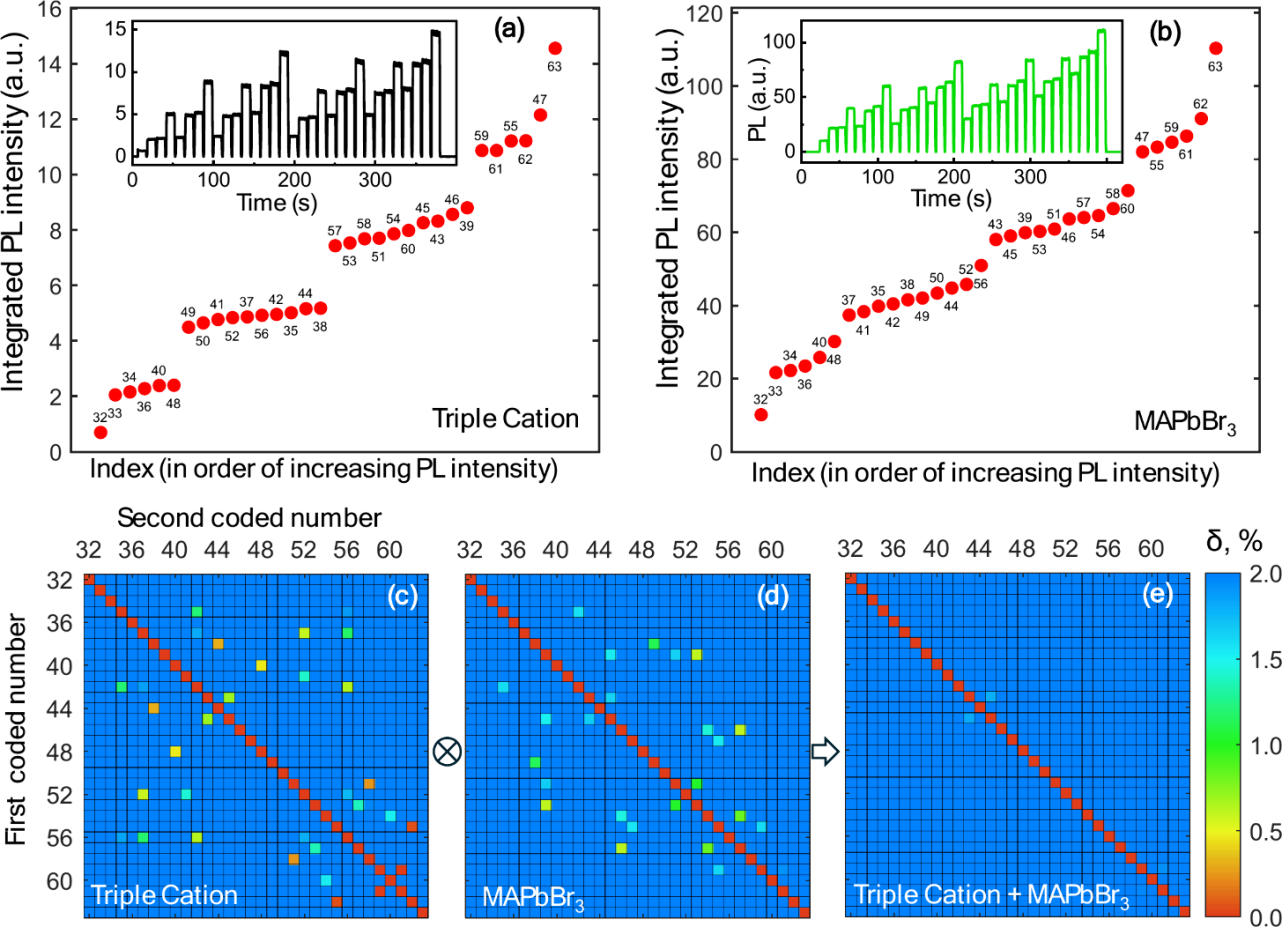
Experimental PL responses of two different perovskite memlumors (triple cation film and MAPbBr_3_ film) to the binary excitation pulse burst sequences encoding the decimal numbers from 32 to 63. a) and b) Integrated PL signal for different binary sequences arranged in ascending order of the time-integrated PL intensity. The insets in a) and b) show the actual readings of the CCD camera in response to the sequentially applied excitation light encoding each binary number from 32 (code "100000") to 63 (code "111111"). c, d) The separation matrices for both memlumors. e) A combined separation matrix when two memlumors are used to solve the task simultaneously. All coded numbers are separated from each other by at least 2%, apart from one pair, where the separation is around 1.5%.

Sequences that are difficult to separate are clearly seen in the separation matrices (Figure 3c,d) calculated for the experimental data in the same way as for the modeled data. Both samples have more than 10 pairs of codes that are insufficiently resolved. However, in agreement with the simulations, these pairs are usually different between the two memlumors. It means that in cases of almost identical outputs for one memlumor, the outputs are sufficiently distinct for the other (Figure S6b). Thus, the combination of these two materials makes it possible to overcome this limitation. The combined separation matrix (Figure 3e) shows that only one such point remains; however, since, at this point, the separation is still larger than 1%, it can be considered acceptable for this first proof-of-concept demonstration. It is additionally illustrated in SI in Figure S5 where signals for all codes are plotted in the 2-dimentional (S_1_,S_2_) space.

The binary code classification scheme presented here is a simple version of a reservoir computer with one time-dependent input and two time-integrated outputs of the reservoir (SI Figure S7). The time-dependent input (laser pulse bursts) is spread over a multidimensional space of charge and trap dynamics in the two memlumors (reservoir), while the time-integrated PL intensities serve as two reservoir outputs. We learned how to interpret these signals experimentally by applying predefined sequences and measuring the reservoir outputs. The result of learning is presented as a set of 32 regions in the two-dimensional (S_1_, S_2_) plane corresponding to unique 5-bit sequences. The output of the reservoir computer is then determined by the region to which the experimental signal data belongs. This interpretation algorithm is the reservoir computer’s third (output) layer as illustrated in Figure S7.

In future works, we envision a more complex setup where multiple spatially distributed memlumors of different properties are used and illuminated simultaneously with a laser beam while multiple detectors measure their spatially resolved (and/or spectrally resolved) responses. Interpreting such complex signals will require further training of the computer output (either *in silico* or/and directly on hardware). Having a more complex reservoir could lead to solving more complicated problems. The rich and diverse photophysics and optical properties of metal halide perovskites make them particularly promising for these advanced applications.

To conclude, we demonstrated the capability of metal halide perovskite films to perform time-dependent binary sequence classification using time-integrated photoluminescence. Charge trapping and photodoping make the photoluminescence quantum yield of metal halide perovskites to depend on the temporal history of the excitation light, allowing these materials to work as memlumors and to classify temporal input signals without requiring time-resolving electronics. The 5-bit encoding benchmark introduced in this study underscores the potential of perovskite memlumors for neuromorphic and optical computing applications. We demonstrated that combining materials with different photophysical properties, such as MAPbBr_3_ and triple cation perovskites, significantly improves binary sequence separation, addressing the limitations of individual samples. This highlights the importance of designing smart hardware architectures that benefit from the material diversity for solving more complex computational problems. Thus, the simplicity of tuning perovskite compositions, their sizes and shapes at the nanoscale are envisioned to be the driving force toward further memlumor architectural improvements.

## Supplementary Material



## Notes

Sample preparation, optical setup, modeling of the PL response using a Shockley-Read-Hall model, signal separation in 2D space (two memlumors) in the presence of errors, practical realization of the binary codes using laser pulse bursts, modeling of the time-resolved response at the conditions used in the experiments with perovskites (PDF)
