# Supplementary material for: Classification of 5‑bit Binary Light Pulse Sequences Using Photoluminescence of Metal Halide Perovskite Memlumors
Source: ACS Energy Lett. 2025 Jul 11;10(8):3729–34. doi: 10.1021/acsenergylett.5c01369 (PMC12340972; doi:10.1021/acsenergylett.5c01369)
Supplement: Supplementary file 1 [file nz5c01369_si_001.pdf]

Supporting information for

Classification of 5-bit Binary Light Pulse Sequences Using Photoluminescence of Metal Halide Perovskite Memlumors

Jitendra Kumar[1§], Daniel Lizotte[1§], Alexandr Marunchenko[1], Dmitry Tatarinov[2], Shivam Singh[3,4], Yana Vaynzof[3,4], Ivan. G. Scheblykin[1*]

[§]J.K. and D.L. contributed equally to this work

*E-mail: ivan.scheblykin@chemphys.lu.se

[1] Chemical Physics and NanoLund, Lund University, 22100 Lund, Sweden

[2] School of Physics and Engineering, ITMO University, St. Petersburg 197101, Russian Federation

[3] Chair for Emerging Electronic Technologies, Technical University of Dresden, 01187 Dresden, Germany,

[4] Leibniz-Institute for Solid State and Materials Research Dresden, 01069 Dresden, Germany

Table of content

1. Sample preparation 2

1.1 Triple cation perovskite films

1.2 $MAPbBr_3$ perovskite films

2. Optical setup 2

3. Calculating PL response of a perovskite memlumor using a Shockley-Read-Hall model 3

4. Practical realization of the binary codes using laser pulse bursts 5

5. Modelling of the PL response of Memlumor_1 and Memlumor_2 to the binary codes 5

6. Signal separation in 2D space (two memlumors) in the presence of errors 6

References 11

1. Sample preparation

1.1 Triple cation perovskite films.

To prepare the triple cation films the precursor solution was prepared by dissolving PbI_2 and $PbBr_2$ in a solvent mixture (DMF/DMSO = 4/1) and CsI in DMSO. CsI solution was heated at 180 °C for 10 minutes. After cooling down, CsI solution, PbI_2 and $PbBr_2$ solutions were mixed in a volume ratio of 0.05:0.85:0.15, to obtain an inorganic stock solution. In two separate vials FAI and MAI powders were added and weighed, into which the appropriate amount (0.95:1 molar ratio) of inorganic stock was added. This creates two new solutions, of the formula $Cs_{0.05}(FA\ or\ MA)_{0.95}Pb(I_{0.9}Br_{0.1})_3$. Finally, these two solutions were mixed in a 5:1 v/v ratio, in order to achieve the final molecular formula $Cs_{0.05}(FA_{0.83}MA_{0.17})_{0.95}Pb(I_{0.9}Br_{0.1})_3$. The perovskite layer was deposited via a two-step spin-coating procedure with 1000 rpm for 12 s and 5000 rpm for 28 s. Before spinning, 40 µL of perovskite precursor solution was applied to the sample statically, and 150 µL of Trifluorotoluene (TFT) was dripped on the spinning substrate, 5 s before the end of the second spin-coating step in a rapid fashion, in agreement with Taylor et al.[1] Subsequently, the samples were annealed at 100 °C for 30 min to form perovskite films.

1.2 $MAPbBr_3$ perovskite films

Materials. Methylammonium bromide (MABr, > 99.99 %, Greatcell Solar Materials), lead (II) bromide ($PbBr_2$, 99.998 % trace metals basis, TCI Chemicals), dimethyl sulfoxide (DMSO, anhydrous, ≥ 99.8 %, Sigma-Aldrich) were used as received.

Preparation of $MAPbBr_3$ precursor solution. To prepare $MAPbBr_3$ perovskite films, we used a perovskite solution with 10 % excess of methylammonium bromide. Powders of MABr (61.58 mg, 0.5 mmol) and $PbBr_2$ (183.5 mg, 0.5 mmol) were mixed and dissolved in 1 ml of DMSO by shaking for 5 min without heating to obtain a clear 0.5 M $MAPbBr_3$ solution with 10 % excess of MABr. The solution was stored in a N_2-filled glove box to avoid exposure to oxygen and moisture. The solution was filtered through a 0.45 µm PTFE syringe adapter right before the perovskite film deposition.

Films preparation. The glass substrates (20 × 20 mm^2) were subsequently cleaned with Na_2CO_3 powder, ultrasonicated in deionized water, acetone and 2-propanol for 5 min, and, finally, exposed to O_3 for 10 min. Thin films of $MAPbBr_3$ with 10 % excess of MABr were prepared by using precursor solution spin-cast at 3000 rpm for 40 s. Toluene was used as an antisolvent after 20 s of the start of the spinning program to obtain films with a thickness of 100 nm. The obtained films were then annealed at 90 °C for 10 min.

Encapsulation. In our experiments, we used the cover glass encapsulation technique to minimize exposure to moisture and oxygen on the obtained perovskite films. Initially, double-sided tape was applied to the surface of a cover glass (15 mm x15 mm size and 0.13 mm thick) in such a way as to form a square with an area ≈ 1 cm^2. This procedure is necessary to create an area of the perovskite film that will remain active for research after the encapsulation process is completed (the tape prevents the epoxy from elbowing and affecting the surface of the perovskite film). After that, cover slips with double-sided tape were placed on the surface of the obtained perovskite films on glass in a glove box under a nitrogen atmosphere. A two-part epoxy was used to complete the encapsulation and sealing process of the perovskite films. Epoxy (the ratio of polyepoxy to hardener is 1:1) was applied at the contact point between the edge of the cover glass and the glass substrate with the perovskite layer to create the active region hermetically protected from external influences. The obtained samples were left in the glove box for 24 hours until the epoxy completely hardened.

2. Optical setup

We used the same optical setup as in our previous publications.[2] The setup is a home-built wide-field fluorescence microscope based on Olympus IX71 inverted microscope. The PL was excited by a 485 nm

diode laser (PicoQuant, with a ≈200 ps pulse width) through a 40X objective lens. The size of the excitation spot was around 30 µm x 30 µm. The laser was controlled by a Sepia Oscillator Module (SOM 828-D, PicoQuant). This allowed generating complex pulse patterns used for PL excitation, as described in detail elsewhere.[2]

3. Calculating PL response of a perovskite memlumor using a Shockley-Read-Hall model

Theoretical modelling was used to demonstrate the ability of halide perovskites to distinguish the 6-bit binary sequences (coded digital numbers from 32 to 63). We used a model of the perovskite semiconductor based on the Shockley-Read-Hall (SRH) charge recombination model, containing one type of trap (electron trap in our case). This model (Figure S1) is often used to describe charge dynamics in perovskites.[3–6]

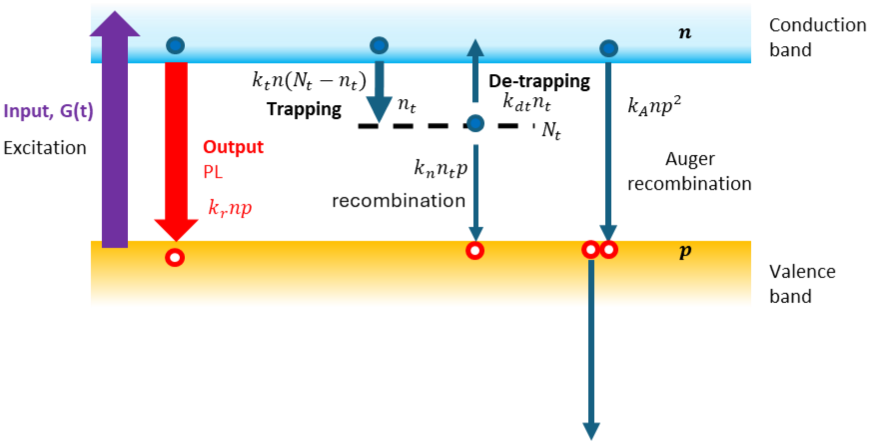

Figure S1. The band diagram showing the processes and their rates in the framework of the Shockley-Read-Hall charge recombination model used for calculations.

The PL intensity is calculated by solving the system of coupled differential equations for electrons (n), trapped electrons (n_t) and holes (p). The system of equations of this model can be written as follows:

$$\frac{d}{dt}n(t) = G - k_t(N_t - n_t)n + k_{dt}n_t - k_r np - k_a np^2 \quad \text{(S3.1)}$$

$$\frac{d}{dt}n_t(t) = k_t(N_t - n_t)\,n - k_{dt}n_t - k_n n_t p \quad \text{(S3.2)}$$

$$\frac{d}{dt}p(t) = G - k_n n_t p - k_r np - k_a np^2 \quad \text{(S3.3)}$$

With the following conditions for the charge photogeneration:

$$G = n_0 \quad \{when\ the\ laser\ pulse\ is\ excpected\} \quad \text{(S3.4)}$$

$$G = 0 \quad \{where\ there\ is\ no\ laser\ pulse\} \quad \text{(S3.5)}$$

Here G is the charge carrier generation per pulse, k_t is the electron trapping rate, N_t is the trap density, k_{dt} is the de-trapping rate, n_t is the trapped electron density, k_r is the radiative recombination rate, k_n is the non-radiative recombination rate, and k_a is the Auger recombination rate.

Solving equations S3.1 – S3.3 using S3.4 and S3.5 we can find the value of n(t) and p(t), thereafter, we can calculate the PL intensity as a function of time:

$$PL(t) = k_r \cdot n(t) \cdot p(t) \tag{S3.6}$$

The integrated signal (which is observed at CCD camera), is obtained by integrating equation S3.6:

$$Integrated\ PL\ intensity_i = k_r \cdot \int_0^T n_i(t) \cdot p_i(t)dt \tag{S3.7}$$

Where the integration goes from zero to T, where T is the repetition period of the binary sequence. Here it is assumed that we are in the "single sequence regime" (analogues to the single pulse regime [2, 3]), meaning that the period T is so long that no memory is retained until the next excitation sequence. In our experiment, it was the case because T was intentionally set as long as 8 ms. Index '*i*' is the decimal equivalent of the binary bit sequence used to excite the PL (from 32 to 63 in our case).

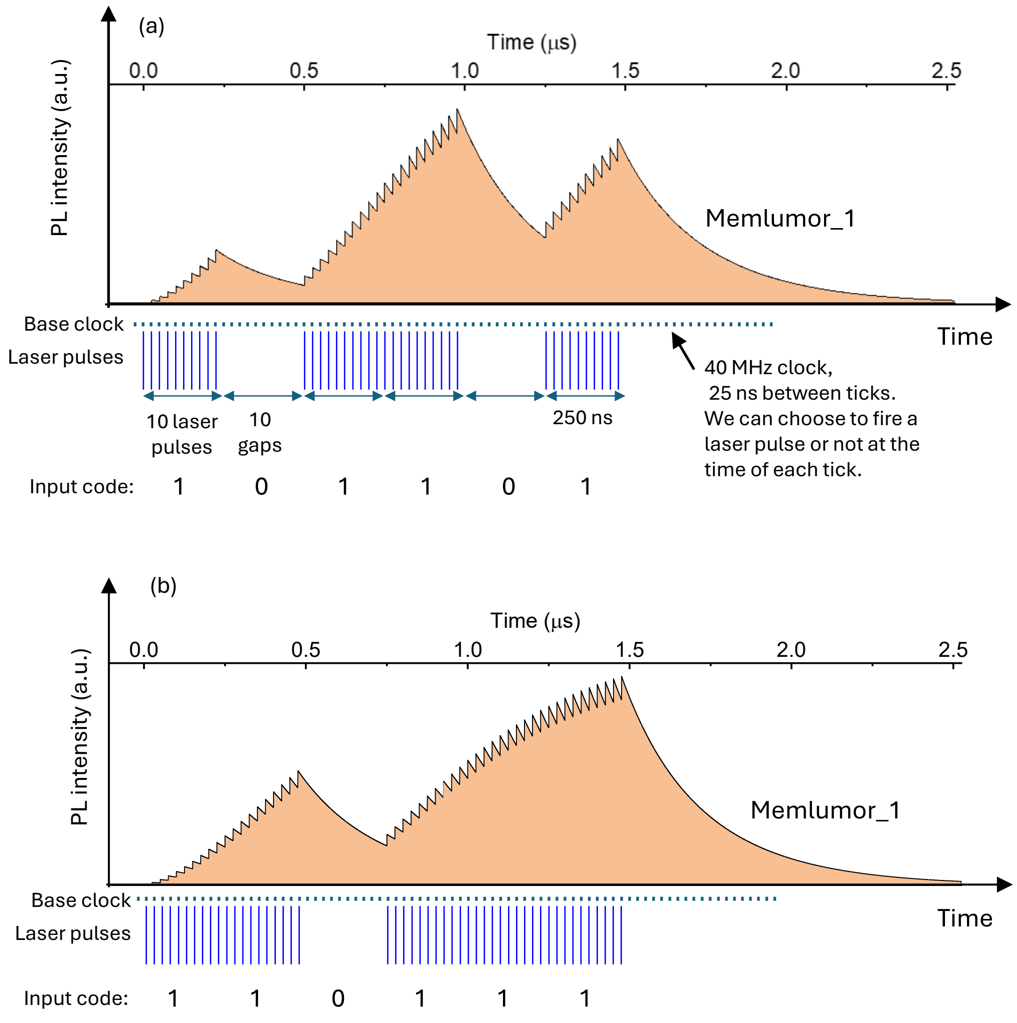

Figure S2. Calculated time-resolved response of Memlumor_1 to two specific binary sequences: (a) – 45, b) - 55. The excitation sequence is organized in the same way as for experiments with perovskite samples: "1" is presented by the bursts of 10 closely spaced pulses (25 ns between pulses), and "0" – by the gap of the same length. Note the dotted timeline, where the dots show ticks of the 40MHz base clock of the laser controller. Using the controller, we can choose to fire a laser pulse or not at each tick of the clock. So, "1" is 10 laser pulses fired in a row, while "0" is 10 ticks without any pulse fired.

4. Practical realization of the binary codes using laser pulse bursts

Due a technical issue related to the slight dependence of the relative amplitude of the pulses in the pulse burst mode on the number of pulses for our particular laser, we could not use just a single pulse as “one” in the binary code as described in the introduction of the paper (Figure 1). Instead, we used a burst of 10 pulses at a repetition frequency of 40 MHz (with 25 ns separation between neighboring pulses, pulse fluence ≈ 17.6 nJ/cm^2) as “one” (Figure S2). One can see the “one” as a quasi-continuous excitation which lasts 250 ns. Then “zero” was just a delay of the same length (absence of the burst), see Figure S2 for all details. Each bit has 250 ns length, thus the 6-bit input sequence lasted 6×250 ns = 1.50 µs and it was repeated with the repetition period T = 8 ms. Such a long repetition period was needed for the memory created by one sequence to decay until the same sequence excites the sample again (similar to the single pulse excitation regime[3]). The PL intensity (output) was integrated over the exposure time (100 ms, or longer) of the CCD camera used as a detector.

We used home-developed Python based software to control Sepia Oscillator Module (SOM 828-D, PicoQuant). The software was programmed to set the parameters to Sepia to generate the required excitation sequence, apply it for 10 seconds, switch the laser off, change the sequence to the next one, wait 1 s and apply the new sequence again for 10 s and so on. The process was repeated until all sequences coding numbers from 32 to 63 were applied, generating a PL signal measured by CCD, as shown in Figure 3 in the main text.

5. Modelling of the PL response of Memlumor_1 and Memlumor_2 to the binary codes.

Using the SRH model described in SI section 3 and the parameters for Memlumor_1 and Memlumor_2 (Table S1 and S2) we can calculate time-resolved responses of the memlumors to all pulse sequences (see SI section 5 about the composition of the pulse sequences) used in the experiments. Figure S2 shows the response of Memlumor_1 to two examples of bit sequences serving also as the illustration of the experimental realization of the pulse coding applied experimentally to the perovskite samples.

Figure S2a shows the input bit sequence ‘101101’ supplied by the laser pulses (represented by vertical blue lines). First, 10 closely separated laser pulses with a period (distance between the pulses) 25 ns are supplied to the sample, the pulse fluence is 17.63 nJ/cm^2, these pulses change the state of the sample and increase the PL intensity. These 10 pulses form the first bit (“1”) of the sequence “101101”. Thereafter, for the next 250 ns, the absence of any laser pulse forms the next bit (“0”) of the same sequence. Following that, a group of 20 pulses (10 + 10 pulses), which comes after 500 ns, corresponds to bits “11”. Next, a gap of 250 ns again represents “0”, and finally, a group of 10 pulses towards the end of the pattern corresponds to the last “1” of the bit sequence “101101”.

Using the same model and the parameters given in Table S1 and Table S2 upon the excitation sequences of the same composition as described above, the results shown in Figure 2 in the main text were calculated.

Table S1. Model parameters for Memlumor_1 (see calculations in Figure 2 and Figure S2):

k_tN_t	**5.80x10^6 s^{-1}**
N_t	**4.36x10^{15} cm^{-3}**
k_{dt}	**0 s^{-1}**
k_n	**4.46x10^{-10} cm^{3}s^{-1}**
k_r	**4.19x10^{-11} cm^{3}s^{-1}**
k_a	**1.68x10^{-28} cm^{6}s^{-1}**

Table S2. Model parameters for Memlumor_2 (see calculations in Figure 2):

k_tN_t	**$1.07x10^8$ s^{-1}**
N_t	**$2.22x10^{15}$ cm^{-3}**
k_{dt}	**$1.24x10^5$ s^{-1}**
k_n	**$5.50x10^{-11}$ cm^3s^{-1}**
k_r	**$2.02x10^{-10}$ cm^3s^{-1}**
k_a	**$5.48x10^{-27}$ cm^6s^{-1}**

6. Signal separation in 2D space (two memlumors) in the presence of errors

When one memlumor is used we calculate the relative difference between the signals $S(i)$ and $S(j)$ generated by the excitation sequences *i* and *j*. We consider the sequences separated when this relative separation is larger than 2δ , where δ is the standard deviation (relative error) of the normalized signal:

$$\delta_{ij} = \frac{(|S(i)-S(j)|)}{S(i)} > 2\delta \qquad \text{(S6.1)}$$

Where in our case the excitation sequences (codes) *i* and *j* range from 32 to 63. This relative separation is plotted as separation matrix for individual memlumors in Figure 2 and Figure 3 the main text.

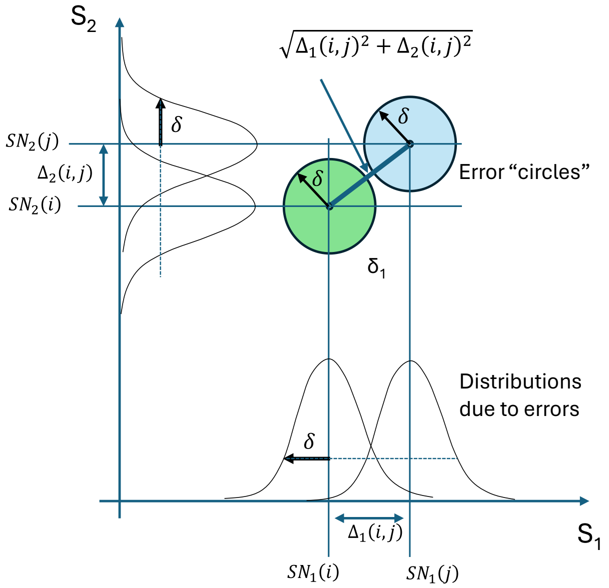

Figure S3. Illustration of how having signals from two memlumors at the same time helps to separate signals created by sequences *i* and *j*. The normalized signals (SN) are plotted. The circles have the radii equal to the standard deviation of the signal distribution. See the text for explanations.

Let's assume that we have two memlumors 1 and 2. The signals generated by the excitation sequences *i* and *j* applied to the memlumors are $S_1(i)$, $S_1(j)$, $S_2(i)$ and $S_2(j)$. For each memlumor, these signals are very close to each other (that is why the excitation sequences *i* and *j* are poorly separated).

Then let's apply normalization by dividing the signals of both memlumors by the signal of the corresponding memlumor for code *i*, then $SN_1(i) = 1$, $SN_1(j) = S_1(j)/S_1(i) \approx 1$ and $SN_2(i) = 1$,

$SN_2(j) = S_2(j)/S_2(i) \approx 1$. Let's assume that the signals have the same relative experimental error δ, *i.e.* the values SN are normally distributed with the standard deviation δ.

Then the separation criteria Eq. (S6.1) between the sequences *i* and *j* for each memlumor separately can be written as:

$$\frac{|S_1(i) - S_1(j)|}{S_1(i)} \cong |SN_1(i) - SN_1(j)| = \Delta_1(i,j) > 2\delta \text{ – the signals are separated by Memlumor 1} \quad \text{(S6.2)}$$

$$\frac{|S_2(i) - S_2(j)|}{S_2(i)} \cong |SN_2(i) - SN_2(j)| = \Delta_2(i,j) > 2\delta \text{ - the signals are separated by Memlumor 2} \quad \text{(S6.3)}$$

When we use two signals at the same time, we need to consider the distance between the points $(SN_1(i), SN_2(i))$ and $(SN_1(j), SN_2(j))$ in the S_1, S_2 -plane, which is equal to:

$$\Delta_{12}(i,j) = \sqrt{\left(SN_1(i) - SN_1(j)\right)^2 + \left(SN_2(i) - SN_2(j)\right)^2} = \sqrt{\Delta_1(i,j)^2 + \Delta_2(i,j)^2} \quad \text{(S6.4)}$$

This distance should be compared with the relative error δ, so the separation criteria will be

$$\sqrt{\Delta_1(i,j)^2 + \Delta_2(i,j)^2} \; > 2\delta \quad \text{(S6.5)}$$

Obviously, this criterion is easier to satisfy than the criteria (S6.2) or (S6.3).

All this is illustrated in Figure S3 where each individual memlumor cannot separate the sequences "i" and "j" because the criteria (S6.1) is not satisfied for both memlumors (error distributions overlap too much), however, when operated together, the separation is achieved according to (S6.5).

Using the considerations above, we characterize separation by two memlumors operating together by the separation matrix $\delta_{1+2,ij}$ calculated as:

$$\delta_{1+2,ij} = \sqrt{{\delta_{1,ij}}^2 + {\delta_{2,ij}}^2} \quad \text{(S6.6)}$$

Where $\delta_{1,ij}$ and $\delta_{2,ij}$ are separation matrices for memlumors 1 and 2, respectively.

To show the advantage of having a combination of two memlumors we plot the PL intensities created by different bit sequences in a 2-dimensional space of PL intensity of Memlumor_1 (x-axis) and Memlumor_2 (y-axis) on Figure S4. The figure shows the advantage of looking at the separation between the PL signals by plotting all the data points together with a 1% error bar. As can be seen from the figure that the ellipses carved by the error bars of 1% do not overlap with each other, which means that all the data points are at least 2% different from each other. Whereas by looking at the projection of the error bars to one of the axes, we can see that the error bars overlap with each other. It means that these data points cannot be separated from each other if the signal is generated from only 1 memlumor.

Figure S4 and Figure S5 below show how the data points obtained from the numerical modeling (Memlumor_1 and Memlumor_2) and from the two memlumors measured experimentally are spread in the S1, S2 plane.

Figure S6 shows the difference between the signals from two memlumors in the case where the data is sorted according to the increase of the output of one of the memlumors.

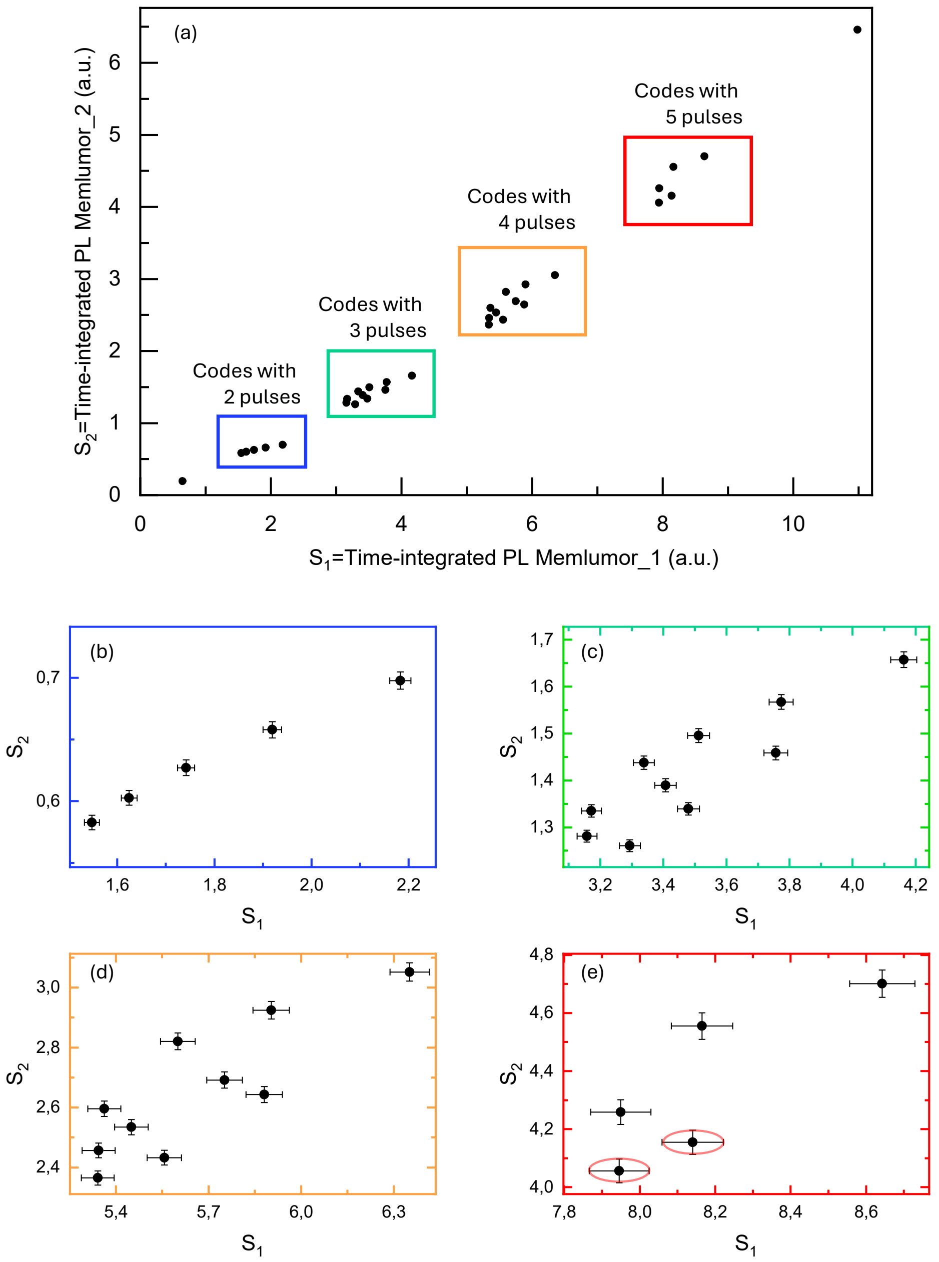

Figure S4. Calculated PL responses of Memlumor_1 and Melumor_2 plotted as points in the S1, S2 plane. (a) Each data point corresponds to one excitation sequence (code) with the coordinates (S1, S2). If two data points are too close to each other, the excitation sequences corresponding to them are poorly separated (can be mixed up). (b), (c), (d, (e) are zooms into the regions of the graph in (a) corresponding to codes with the same number of pulses (2, 3, 4 and 5, respectively). Errors bars are 1 % for each axis. As one can see, the separation between the data points is always larger than the error bar. In the panel (e) the ellipses show how the error distribution looks like in 2D plane (the axes or the ellipse are standard deviations for each signal).

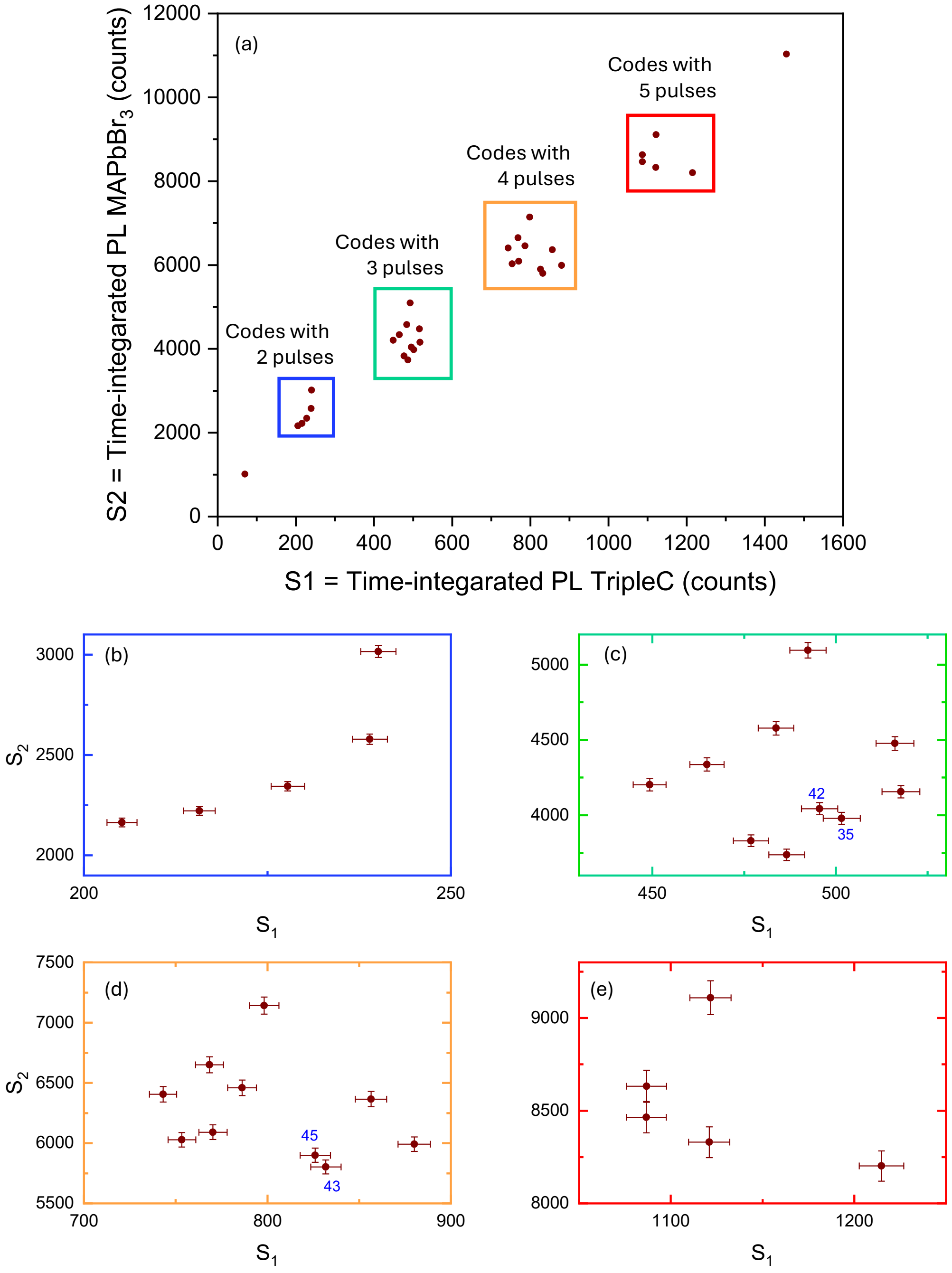

Figure S5. Measured PL response of $MAPbB_3$ and triple cation perovskite memlumors plotted as points in the S1, S2 plane. (a) Each data point corresponds to one excitation sequence (code) with coordinates (S1, S2). If the data points are too close to each other, the excitation sequences corresponding for them are poorly separated (can be mixed up). (b), (c), (d, (e) are zooms into the regions of the graph in (a) corresponding to codes with the same number of pulses (2, 3, 4 and 5, respectively). Errors bars are 1 % for each axis. As one can see, the separation between the data points is always larger than the error bars. Some closely located points are marked with their codes in (c) and (d).

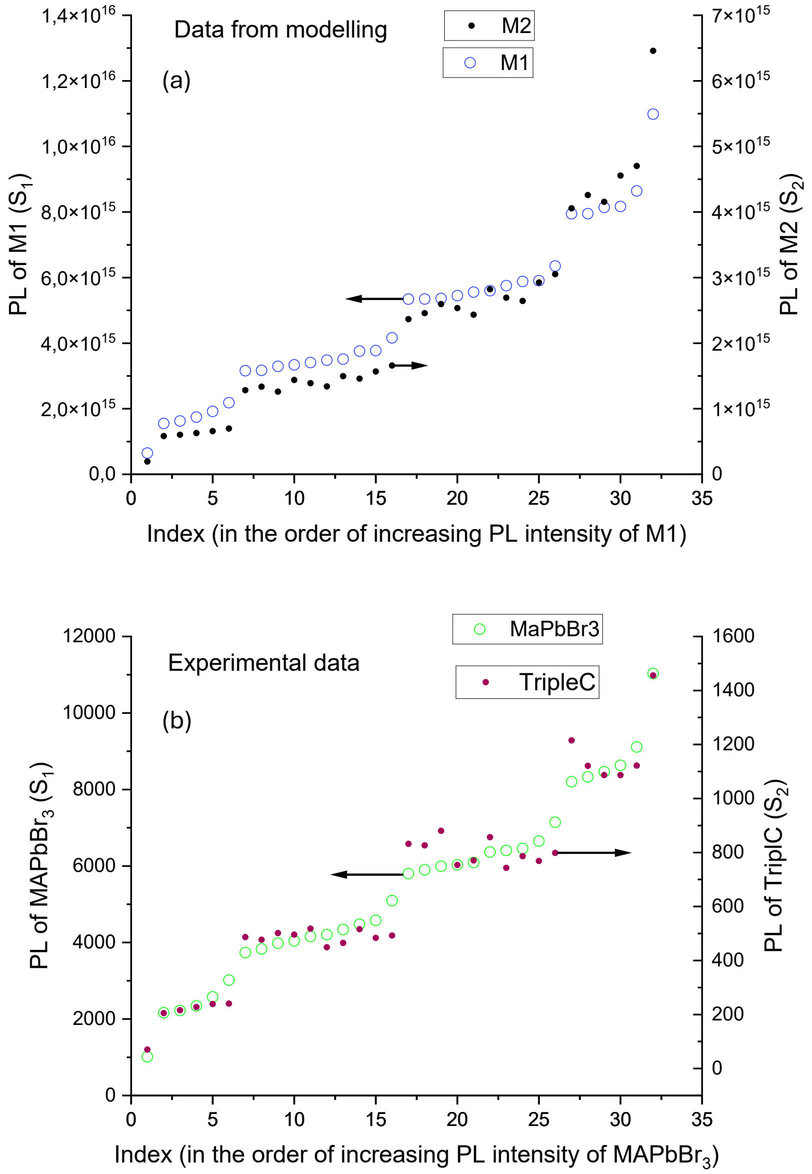

Figure S6. Comparison of PL responses of (a) modelled Memlumor_1 and Memlumor_2 and (b) $MAPbB_3$ and triple cation perovskite memlumors. The data are sorted according to the increasing of PL intensity of one of the memlumor from each couple. The neighboring data points should have different PL values to be separated. One can see that where the difference in the responses of one memlumor is small, the difference in the responses of the other memlumor is sufficient for separation.

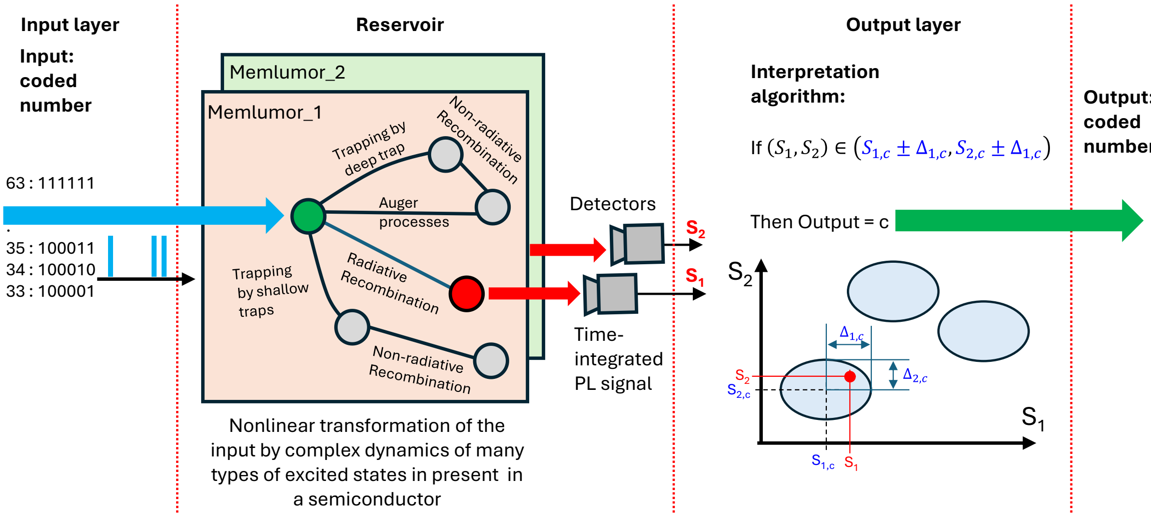

Figure S7. Schematic presentation of the classification setup as a luminescence-based reservoir computer.

References

(1) Taylor, A. D.; Sun, Q.; Goetz, K. P.; An, Q.; Schramm, T.; Hofstetter, Y.; Litterst, M.; Paulus, F.; Vaynzof, Y. A General Approach to High-Efficiency Perovskite Solar Cells by Any Antisolvent. *Nat Commun* **2021**, *12*, 1878 https://doi.org/10.1038/s41467-021-22049-8.

(2) Marunchenko, A.; Kumar, J.; Tatarinov, D.; Pushkarev, A. P.; Vaynzof, Y.; Scheblykin, I. G. Hidden Photoexcitations Probed by Multipulse Photoluminescence. *ACS Energy Lett* **2024**, *9* (12), 5898–5906. https://doi.org/10.1021/acsenergylett.4c02404.

(3) Kiligaridis, A.; Frantsuzov, P. A.; Yangui, A.; Seth, S.; Li, J.; An, Q.; Vaynzof, Y.; Scheblykin, I. G. Are Shockley-Read-Hall and ABC Models Valid for Lead Halide Perovskites? *Nat Commun* **2021**, *12*, 3329. https://doi.org/10.1038/s41467-021-23275-w.

(4) Marunchenko, A.; Kumar, J.; Kiligaridis, A.; Rao, S. M.; Tatarinov, D.; Matchenya, I.; Sapozhnikova, E.; Ji, R.; Telschow, O.; Brunner, J.; Yulin, A.; Pushkarev, A.; Vaynzof, Y.; Scheblykin, I. G. Charge Trapping and Defect Dynamics as Origin of Memory Effects in Metal Halide Perovskite Memlumors. *J Phys Chem Lett* **2024**, *15* (24), 6256–6265. https://doi.org/10.1021/acs.jpclett.4c00985.

(5) Yuan, Y.; Yan, G.; Dreessen, C.; Rudolph, T.; Hülsbeck, M.; Klingebiel, B.; Ye, J.; Rau, U.; Kirchartz, T. Shallow Defects and Variable Photoluminescence Decay Times up to 280 Ms in Triple-Cation Perovskites. *Nat Mater* **2024**, *23*, 391–397. https://doi.org/10.1038/s41563-023-01771-2.

(6) Trimpl, M. J.; Wright, A. D.; Schutt, K.; Buizza, L. R. V.; Wang, Z.; Johnston, M. B.; Snaith, H. J.; Müller-Buschbaum, P.; Herz, L. M. Charge-Carrier Trapping and Radiative Recombination in Metal Halide Perovskite Semiconductors. *Adv Funct Mater* **2020**, *30* (42), 2004312. https://doi.org/10.1002/adfm.202004312.
